# A Preach to Preachers

**Published:** 1871-09

**Authors:** 


### A PREACH TO PREACHERS.

All apologies are founded in self-deception or pride.

Move directly to your subject; the sooner you get at the main idea, the better.

One telling illustration is better than a dozen, even if each of the dozen are quite as strong.

Let the subject be of such importance as to invest you with an inspiration, until the closing sentence.

Encourage, rather than scold.

Do not make many points; two or three well insisted on will be longer remembered than half a dozen.

Never think of yourself, but of a soul saved or lost, and Calvary.

Have only two or three headings and one application — clear, short, and to the point, so that it may still be ringing in the ears of the people as they are dispersing.

If you cannot preach from a text without an intimation that a different translation would improve it, select some other passage, or a different subject.

Feel that this sermon may be your last.

